# Analysis of Proteins Associated with Quality Deterioration of Grouper Fillets Based on TMT Quantitative Proteomics during Refrigerated Storage

**DOI:** 10.3390/molecules24142641

**Published:** 2019-07-20

**Authors:** Xicai Zhang, Jing Xie

**Affiliations:** 1 College of Food Science & Technology, Shanghai Ocean University, Shanghai 201306, China; 2 Shanghai Engineering Research Center of Aquatic Product Processing and Preservation, Shanghai 201306, China; 3 Shanghai Professional Technology Service Platform on Cold Chain Equipment Performance and Energy Saving Evaluation, Shanghai 201306, China; 4 National Experimental Teaching Demonstration Center for Food Science and Engineering Shanghai Ocean University, Shanghai 201306, China; 5 College of Bioengineering, Jingchu University of Technology, Jingmen 448000, China

**Keywords:** TMT, proteomics, grouper fillets, quality deterioration, refrigerated storage

## Abstract

A TMT (Tandem Mass Tag)-based strategy was applied to elucidate proteins that change in proteomes of grouper fillets during refrigerated storage. In addition, quality analyses on pH, centrifugal loss, color (L *, a *, b *) and texture (hardness, chewiness, and gumminess) for grouper fillets were performed. A total of 64 differentially significant expressed proteins (DSEPs) were found in the results in the Day 0 vs. Day 6 group comparison and the Day 0 vs. Day 12 group comparison. It is worth mentioning that more proteome changes were found in the Day 0 vs. Day 12 comparisons. Bioinformatics was utilized to analyze the DSEP. UniProt Knowledgebase (UniProtKB), Gene Ontology (GO) enrichment, Kyoto Encyclopedia of Genes and Genomes (KEGG) and protein interaction network analysis were adopted. All DSEPs were classified into seven areas by function: binding proteins, calcium handling, enzymes, heat shock protein, protein turnover, structural proteins and miscellaneous. The numbers of proteins that correlated closely with pH, centrifugal loss, color (L *, a *, b *) and texture (hardness, chewiness, and gumminess) were 4, 3, 6 and 8, respectively.

## 1. Introduction

Grouper is widely distributed throughout the coastal area of east China. In China, with the rapid development of grouper breeding technology, the annual output exceeds 100,000 tons [1]. Grouper has high crude protein contents and is rich in fatty acids and polyunsaturated fatty acids [2]. Freshness is one of the most important factors for fish because of its strong relationship to nutrition and health; it is essential for food safety to produce high-quality products [3]. However, grouper can easily decompose due to its abundant nutrition and high water content as well as excellent protease activity. As the main method of processing fish, however, freezing preservation may negatively affect sensory quality such as taste [4]. Therefore, refrigerated storage still has wide application in fish circulation, accounting for 45% of the total amount consumed [5]. Protein is one of the most important nutrients in aquatic products, which play a major role in quality parameters such as texture and color [6].

More recently, proteomics has been widely applied in the field of food production processes as well as in food safety, as a powerful tool for unraveling the mechanisms of biochemistry between muscle proteins and quality traits [7]. Two-dimensional electrophoresis (2-DE) coupled with mass spectrometry (MS) has been frequently used for proteomics analysis and successfully applied in many previous studies [8,9,10]. However, the method of 2-DE coupled with MS has many defects in protein identification, i.e., proteins that are too large or small might be hard to recognize. Moreover, extremely hydrophobic and low abundance proteins are also difficult to recognize [11].

With the development of proteomic technologies, high-resolution and high-throughput mass spectrometry has evolved into a mature technology [12]. As one of the most robust proteomics techniques, TMT (Tandem Mass Tags) is a kind of relative and absolute quantitative techniques of isotopic labeling in vitro [6,13], which was developed by Thermo Scientific (Thermo Fisher Scientific, USA). TMT can identify and quantify proteins from 10 different samples by high-resolution mass spectrometer series analysis at the same time. It can reduce the errors caused by experimental measurement. TMT is mainly used in physiological and pathological research for animals and plants [14,15,16]. However, application of TMT to study total proteome changes and molecular mechanisms of grouper fillets during refrigerated storage has not been reported.

In this study, the proteome changes of grouper fillets after 0, 6, and 12 days of refrigerated storage were obtained by TMT-coupled LC-MS/MS, and potential biochemical markers were identified from the experimental data. Correlation analysis between differential proteins and quality index revealed the underlying mechanisms of quality deterioration, providing a new view at a deeper level in grouper fillets during refrigerated storage.

## 2. Results and Discussion

### 2.1. Quality Change of Grouper Fillets

The pH, centrifugal loss, color, and texture, as important quality evaluation indices of grouper muscle, were evaluated. The results are shown in Table 1.

Comparing the data from Day 0, Day 6 and Day 12 samples, pH and centrifugal loss were increased along with prolonged time during storage, which was similarly observed for carp (*Cyprinus carpio*) fillets during storage [17,18]. The reason for the increase in pH might be due to the accumulation of amine and ammonia caused by the degradation of proteins and microbial growth. The increasing of centrifugal loss (*p* < 0.05) might be attributed to the decrease of water holding capacity of grouper fillets because of the denaturation and aggregation of the myofibrillar proteins [17]. The L * of color declined significantly, which might be due to fish browning with the storage time. The a * and b * values increased during storage. It is reported that blood hemoglobin and myoglobin histone react with oxygen, which leads to a decline in fish color [19]. Texture is one of the important sensory indices for grouper fillets. The texture of grouper changed because of the degradation of major structural proteins. Hardness, chewiness, and gumminess decreased significantly (*p* < 0.05) in both Day 6 and Day 12 groups compared with the Day 0 group.

### 2.2. Identification of Proteins by Quantitative Proteomics Analysis

**Figure 1:**
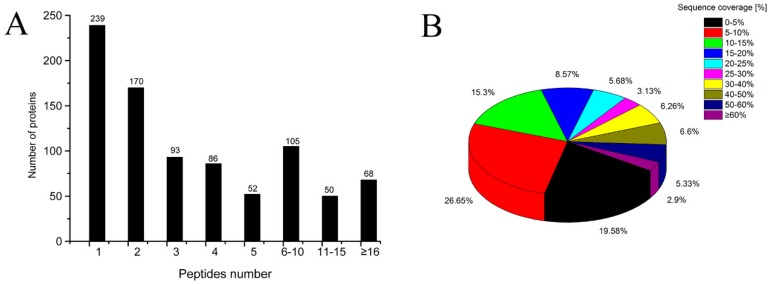
(**A**) Number of peptides matched to proteins; and (**B**) percentages of sequence coverage of proteins by identified peptides.

Spectrometric analyses in this study included protein identification, peptide identification, protein quantification, and differential protein classification analysis, among others. We obtained 21,213 peptide spectrum matching (PSM) numbers and 4649 unique peptides, which were mapped to 863 proteins with 817 being quantified as proteinases after data analysis (Table S1). Nearly 28% of the identified proteins contained a single peptide (Figure 1A). The results show that the protein recognition sequence had a high coverage rate, with more than 10% of the sequence coverage ratio reaching 53.77% of the protein number (Figure 1B).

### 2.3. General Information for TMT-LC-MS/MS Analysis

**Figure 2:**
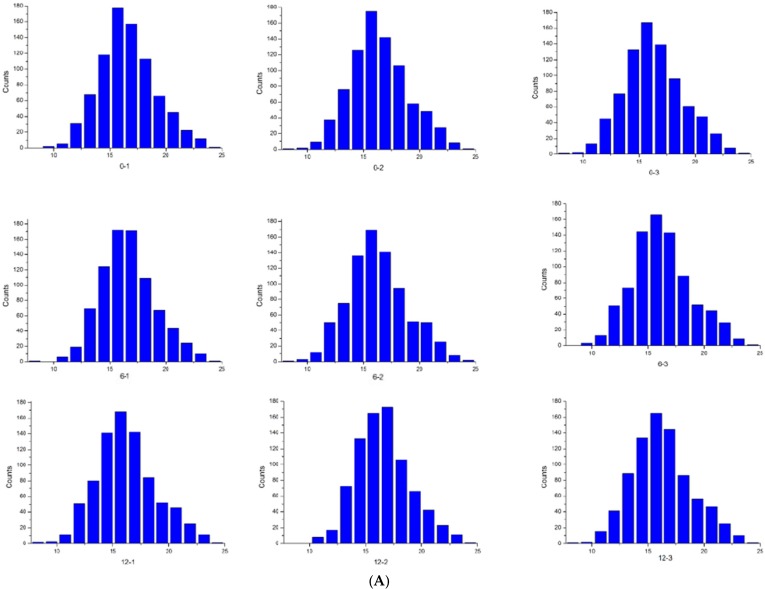

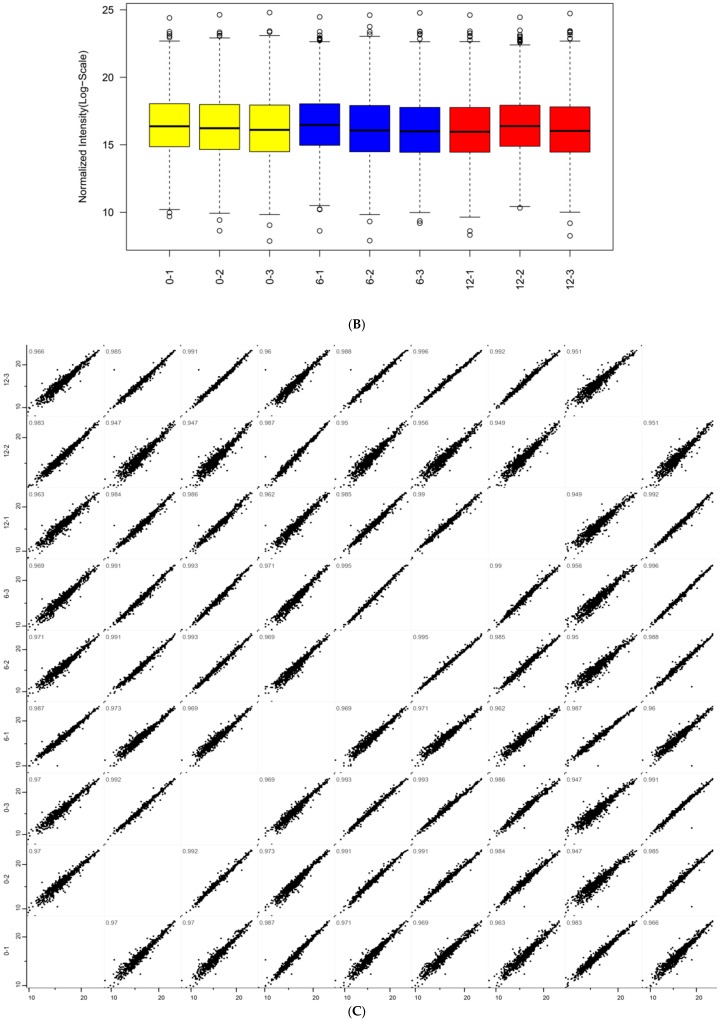
(**A**) Histogram of log2 protein intensity for each Sample; (**B**) box plots of log2 protein (or Reporter ion) intensity average for each sample; and (**C**) a matrix of scatter plots and Pearson correlation coefficient of Protein intensities for each sample.

A histogram of intensity for each sample is shown in Figure 2A. Figure 2B shows a box plot of normalized density, i.e., box plots of log2 protein intensity average for each sample. Pearson correlation coefficients of the nine samples (three parallel samples for each group) show high repeatability through the evaluation of relative quantitative protein (R > 0.95) (Figure 2C).

### 2.4. Identification of Differentially Significant Expressed Proteins (DSEPs)

**Figure 3:**
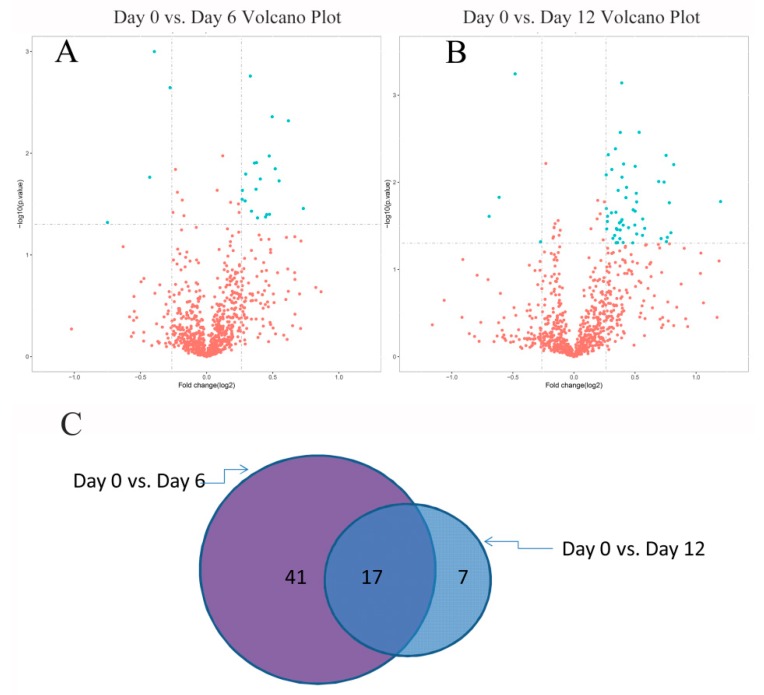
(**A**) Volcano plot of grouper fillets on Day 0 vs. Day 6; and (**B**) volcano plot of grouper fillets on Day 0 vs. Day 12. The abscissa is multiples for difference (logarithmic transformation of 2 at the bottom), while the ordinate is significant difference (*P* value, logarithmic transformation of 10 at the bottom). Green Blue points indicate differentially significant expressed proteins (DSEPs) (fold-change of >1.20 or <0.83 and *p*-values < 0.05). (**C**) The overlap of DSEPs from the Day 6 vs. Day 0 and Day 12 vs. Day 0 comparisons.

As shown in the volcano plots (Figure 3A,B), compared with the Day 0 group, a total of 65 proteins were changed significantly in the Day 6 and Day 12 groups. DSEPs were identified when the following two conditions held: fold-change of >1.20 or <0.83 and *p*-values < 0.05. Sixty-five DSEPs were filtered in the Day 6 and Day 12 groups. There were 58 DSEPs and 24 DSEPs for Day 6 vs. Day 0 and Day 12 vs. Day 0, respectively, including 17 shared DSEPs (see Figure 3C) (for more details, see Table S2), where up-regulated proteins and down-regulated proteins are highlighted in red and green, respectively). Thus, there were more protein changes with prolonged storage time. These 24 DSEPs might be potential biomarkers for further quality changes in grouper fillets stored at 4 °C for six days, while all 58 DSEPs might be potential quality biomarkers on Day 12.

**Figure 4:**
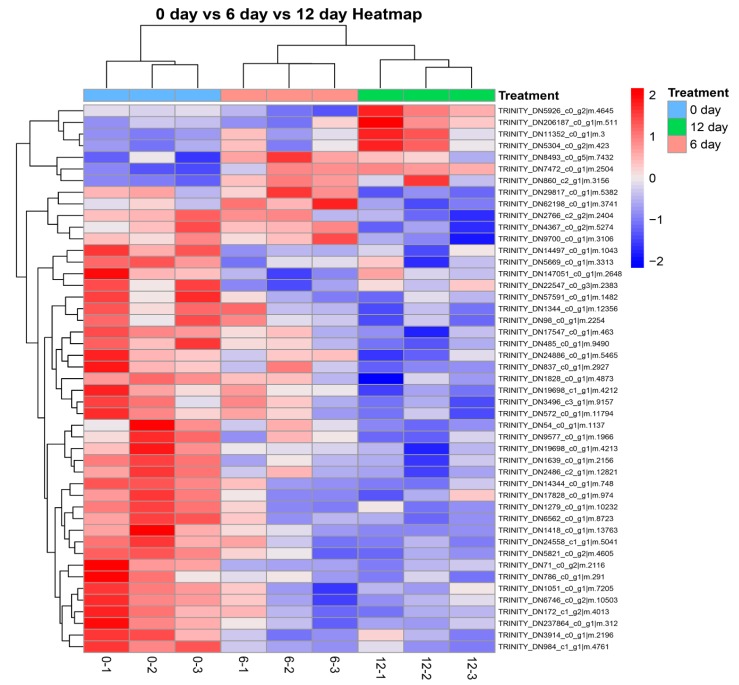
Hierarchical cluster analysis of differentially significant expressed proteins (DSEPs) in grouper fillets on Day 6 and Day 12 compared with Day 0. The right side of each corresponding row is the protein’s Uniprot number. Different colors represent the different relative abundance of proteins (lower intensity are shown in blue and red represents higher intensity).

Clustering algorithms classify two dimensions, namely sample and variable, which refer to the quantitative information of proteins. The results of clustering are shown in Figure 4. Variable, which refers to the quantitative information of proteins, was classified in two dimensions. The clustering results of target proteins can help us to distinguish protein subsets with different expression patterns from protein sets. Proteins with similar expression patterns may have similar functions or participate in the same biological pathway. Similar color distributions indicate similar changes in the trend of proteins that were seen on Day 0 vs. Day 6 of Day 0 vs. Day 12. The same method was used to analyze proteomics data on largemouth bass (*Micropterus salmoides*) fillets by Cao et al. [20]. We found some differences among the three replicates in color for each group, which might be due to individual differences of grouper fillets.

### 2.5. Bioinformatic Analysis of Differentially Significant Expressed Proteins (DSEPs)

#### 2.5.1. GO Enrichment Analysis

**Figure 5:**
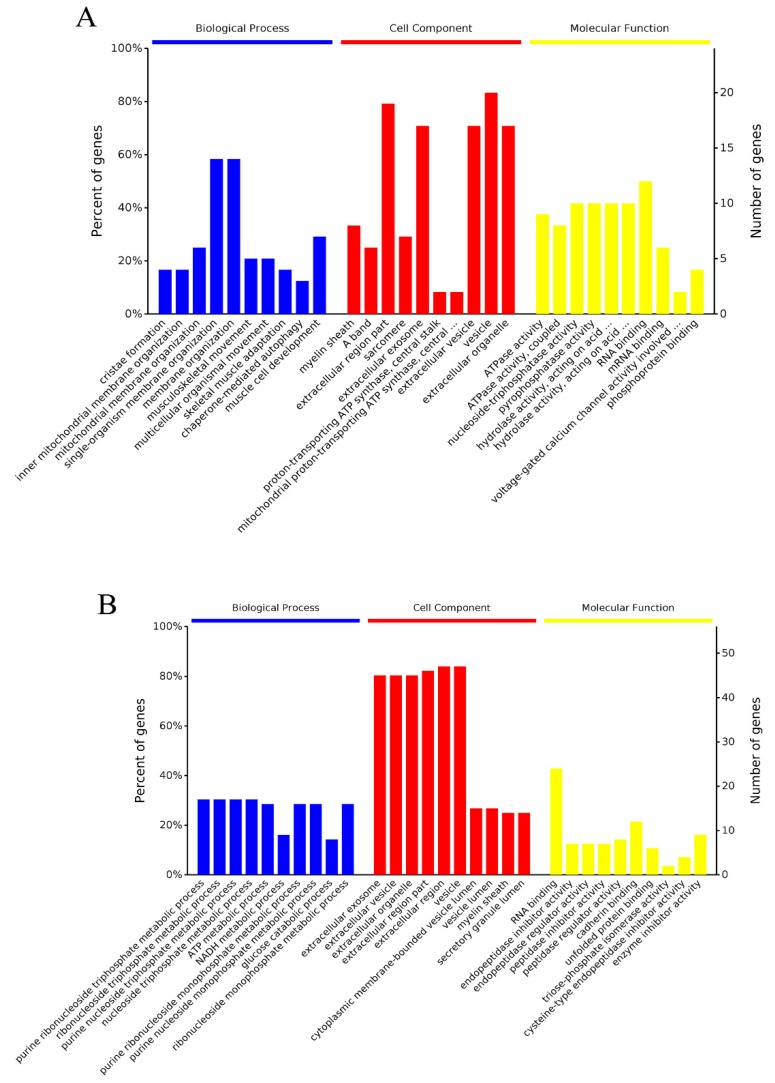
Gene Ontology (GO) enrichment analysis of differentially significant expressed proteins (DSEPs): (**A**) Day 0 vs. Day 6; and (**B**) Day 0 vs. Day 12. The x-axis represents the GO terms, while the y-axis represents the number of DSEPs in each category.

Gene Ontology (GO) is a standardized gene function classification system, which provides a set of dynamically updated standard-controlled vocabulary. It represents three important aspects of biological functions: biological process, molecular function and cellular component [21]. The most enriched 10 terms of GO enrichment analysis for Day 0 vs. Day 6 and Day 0 vs. Day 12 are shown in Figure 5A,B respectively.

For the 24 DSEPs between Day 0 and Day 6, proteins were mainly distributed in inner mitochondrial membrane organization and musculoskeletal movement, under the category of biological process, possibly related to protein hydrolysis. For the cellular component group, macromolecular complex, cytoskeleton, and organelles were also found. In the molecular function group, proteins such as ATPase activity, pyrophosphatase activity, hydrolase activity and mRNA binding were mostly involved. For the 58 DSEPs between Day 0 and Day 12, more proteins for the ribonucleoside triphosphate metabolic, nucleoside triphosphate metabolic and ATP metabolic processes were involved. Proteins with the cellular component classification were similar to the results of the Day 0 and Day 6 comparison. RNA binding, endopeptidase, peptidase, and triose-phosphate isomerase were involved mostly in the molecular function classification. These results are similar to those ones reported for frozen mud shrimp by Shi et al. [11]. More details are presented in Table S3 and S4. Table S3 and S4 show the result of bioinformatics analysis of differentially significant expressed proteins for Day 0 vs. Day 6 and Day 0 vs. Day 12 comparison, respectively.

#### 2.5.2. KEGG Pathway Analyses

**Figure 6:**
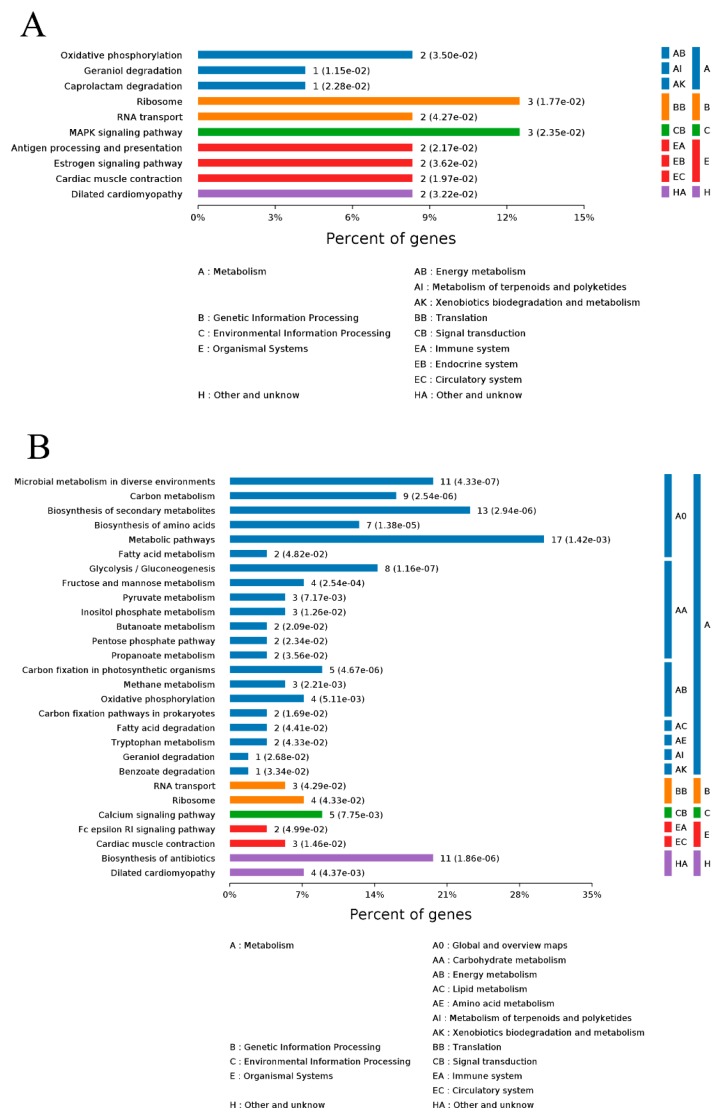
KEGG pathway analyses of differentially significant expressed proteins (DSEPs): (**A**) Day 0 vs. Day 6; and (**B**) Day 0 vs. Day 12.

The KEGG statistics from the significant enrichment analyses on Day 0 vs. Day 6 and Day 0 vs. day 12 are shown in Figure 6A,B, respectively.

The KEGG pathway enrichment analysis method is similar to the GO enrichment analysis but the KEGG pathway is used as the unit. With all qualitative proteins as the background, Fisher’s Exact Test was used to analyze and calculate the significance level of protein enrichment in each pathway to determine the metabolic and signal transduction pathways significantly affected. The DSEPs of Day 0 vs. Day 6 were mainly enriched in the MAPK signaling pathway (map04010), ribosome pathway (map03010), geraniol degradation pathway (map00281) and caprolactam degradation pathway (map00930). We found the DSEPs of Day 0 vs. Day 12 were mainly enriched in glycolysis pathway (map00010), carbon metabolism pathway (map01200), and microbial metabolism in diverse environments pathway (map01120) (Table S5). Activation of MAPK might be cellularly related to apoptosis [22]; the glycolysis pathway, which produces lactate, is the major metabolic pathway after the death of an animal [23].

#### 2.5.3. DSEP Interaction Network Analysis

Proteins do not exist independently and they function with the aid of other proteins. It is of great significance to reveal the qualitative protein features by the formation of a protein interaction network.

**Figure 7:**
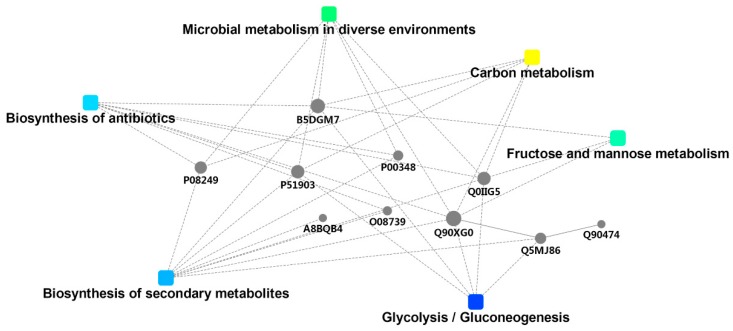
Interaction network analysis of differentially significant expressed proteins (DSEPs) for Day 0 vs. Day 6 vs. Day 12.

As shown in Figure 7, the proteins were mainly associated with carbon metabolism, microbial metabolism in diverse environments, biosynthesis of secondary metabolites, biosynthesis of antibiotics, glycolysis, and fructose and mannose metabolism (for detailsm, see Figure S1). Q90XG0, Q5MJ86, P51903, and Q90474, which are all related to glycolysis, had a relatively high degree of connectivity. B5DGM7 (Fructose-bisphosphate aldolase A) might be the key point of the whole metabolic system due to the high degree connection.

### 2.6. Correlations between Differentially Significant Expressed Proteins (DSEPs) and Quality Index

Pearson correlation analysis was performed between the 65 DSEPs and the quality indices (pH, L *, a *, b *, hardness, chewiness, and gumminess) of grouper fillets during refrigerated storage (Day 6 and Day 12 compared with Day 0). The significant correlations are presented in Table 2 and all details are shown in Table S6. The DSEPs were grouped into six categories according to specific functions: binding proteins, calcium handling, enzymes, protein turnover, heat shock protein family, structural proteins and miscellaneous. The * indicates that a protein has a significant correlation with a quality trait (*p* < 0.05), while ** indicates a highly significant correlation (*p* < 0.01).

### 2.7. Potential Protein Markers Associated with the Quality Change of Grouper Fillets Refrigerated Storage

#### 2.7.1. Binding Proteins

P50143 (T-complex protein 1 subunit gamma) is a kind of chaperonin with the function of folding various proteins [24]. It helps the structure of proteins to remain stable. P50143 was related to the color of grouper fillets significantly, possibly due to the decrease of T-complex protein 1, which led to unstable myoglobin. As shown in Table 2, P06238 (Alpha-2-macroglobulin) was correlated significantly to pH and color. Alpha-2-macroglobulin is a protease inhibitor, which widely distributes in all kinds of animals [25,26]. A negative correlation between Alpha-2-macroglobulin and pH was found. The reason was that, with the decrease of macroglobulin, the activity of protease was increased, which promoted the degradation of protein, leading to the rise of PH value. It has been reported that this protein is closely related to seawater fish [25]. This kind of protein is mainly distributed in the grouper’s blood and can represent the hemoglobin left in the muscle, thus its content is also highly related to the color of the grouper’s flesh.

P20810 (Calpastatin) is a specific, endogenous inhibitor of the calpains. Calpains are widely studied in myofibrillar protein degradation, which leads to softening of muscle [27]. From the analysis in Table 2, calpastatin was significantly related to the texture, e.g. hardness and chewiness, which might be because the activity of calcium protease accelerates with the disappearance of calpastatin, which promotes the myofibril protein degradation. Similar results were reported in previous research [28,29]. The microstructure was changed in the original structure of the muscle because of protein hydrolysis through calcium protease and another protease. The color of muscle, which might be related to light scattering, may be affected by changes of the microstructure [30]. Q3MHM5 (Tubulin beta-4B chain) exerted a significant effect on the centrifugal loss and texture, however, its mechanism is not clear. As an important enzyme of glycolysis, P51903 (Phosphoglycerate kinase) was significantly associated with centrifugal loss. The main changes in muscle were related to enzymes including phosphoglycerate kinase during storage [31]. The level of glycolysis affects the quality and structure of muscle directly.

Q6PHG2 (Hemopexin) has been indicated to promote the progress of lipid oxidation for fish muscle [32]. The oxidation of unsaturated fatty acids could cause deterioration of muscle quality such as nutritional value decreased, odors and discoloration [33], which might be why hemopexin was significantly related to b * value.

#### 2.7.2. Metabolic Enzyme

Q90XG0 (triose-phosphate isomerase) plays an important role in glycolysis. It is indispensable for effective energy generation. The reversible interconversion of dihydroxyacetone phosphate (DHAP) and glyceraldehyde phosphate is catalyzed by triose-phosphate isomerase [34]. Fish muscle postmortem metabolism, which includes glycolysis, could affect meat texture; for example, it could results in softening and increased gaping [23]. Therefore, triose-phosphate isomerase was closely related to hardness. P08249 (Malate dehydrogenase, mitochondrial) is also one of the most important enzymes of glycolysis and plays a key role in many metabolic pathways. We found that the change of malate dehydrogenase could influence the color of grouper fillets such as a * and b *. Previous research proved that malate dehydrogenase was correlated significantly with the a * value and color stability [11,35,36]. Besides, malate dehydrogenase, an oxidoreductase, plays a role in the transport of reductive equivalents between cytosol and mitochondria and keeping the balance of NHDH/NAD^+^ in the mitochondria.

Q0IIG5 (ATP-dependent 6-phosphofructokinase) is one of the rate-limiting enzymes for glycolysis. Its main function is to catalyze fructose 6-phosphate (Fru-6-P) to fructose 1, 6-bisphosphate [37]. Lactate is generated during postmortem metabolism by glycolysis, which affects the pH of the muscle, thus the ATP-dependent 6-phosphofructokinase plays an important role in pH changes. Moreover, the preslaughter muscle concentration of glycogen is considered to decide the ultimate value of pH [38].

#### 2.7.3. Heat Shock Protein (HSP) Family

As molecular chaperones, heat shock proteins play important roles in protein folding and biosynthesis [39]. Q9I8F9 (Heat shock 70 kDa protein 1) was related to gumminess of grouper fillets in this study. The HSP protein family is reported to play an important role in keeping the stable quality of muscle [40]. HSP 70 was produced by the stimulation of cell damage, and it helped to maintain the sarcomere organization through preventing the oligomerization of apoptotic protease activating factor-1 and maturation of caspase-9 [41]. There are also reports that Hsp proteins prevent calpains to efficiently exert their proteolytic activity through binding to damaged myofibrillar proteins [42]. As a result, HSP family proteins played active roles in improving the quality of grouper fillets during refrigerated storage.

#### 2.7.4. Protein Turnover

The ribosome is composed of a small 40S subunit and a large 60S subunit, which is one of the most complex organelles. Ribosomal protein affects the efficiency and stability of tRNA molecule transcription [43]. It has been reported that the 60S ribosome protein L29 purified from the gills of *Crassostrea gigas* and the gill of Pacific oyster (*Pacific oyster*) has antibacterial effects [44], while the mechanism of the 40S ribosome (Q08699) and 60S ribosome (Q90YT6) affecting fish color and pH respectively is still unclear.

#### 2.7.5. Structural Proteins

Five Structural proteins were defined as DSEPs and four of them were significantly correlated with quality indicators of grouper fillet: A2ASS6 (Titin), Q90339 (myosin heavy chain), P23239 (desmin) and A7E2Y1 (myosin-7B).

Titin is a flexible protein. It has a high molecular weight, about 3000 kDa. Titin is an important part of the skeletal muscle fibers filament structure. It accounts for about 8–12% of the total myofibril muscle-associated proteins. To maintain the integrity and stability of myofibril, it connects coarse myofilaments with Z-lines and plays a number of important roles in muscle assembly and elasticity [45]. Table 2 shows that Titin was positively correlated with hardness and chewiness of grouper fillet.

Myosin is a protein of about 500 kDa, consisting of six subunits. With two myosin heavy chains (MHC) and four myosin light chains (MLCs), the composition of the myosin heavy chains may be the most important factor ib many postmortem biochemical processes affecting meat quality, especially pH, color, and tenderness [46]. A previous study showed that the degradation of myosin-1 leads to the variation of a * value in beef muscle during storage [47], which is consistent with our experimental results (Table 2).

Desmin is a substrate of calpain, which is important for cell integrity and muscle function, and was of great significance in the study of meat texture. Richardson demonstrated that reducing noggin degradation generally improves the water holding capacity of meat products [48]. Table 2 shows that desmin was closely related to the hardness of grouper fillet, possibly due to desmin degradation during storage. I Starkey et al. found desmin to be related to the texture of sheep biceps, and it was determined that desmin was affected by nodal protein degradation, although possibly indirectly [49,50].

## 3. Materials and Methods

### 3.1. Sample Preparation

Living grouper (*Epinephelus fuscoguttatus*) (500 ± 50 g) were bought from a local seafood market in Shanghai. All fish were transported to the laboratory immediately and killed within 2 h. The grouper were gutted and washed. Then, they were placed in sterile bags after being drained for 5 min and filleted. All samples were stored at 4 °C and these samples were taken for analysis on Days 0, 6 and Day 12. The fillet samples for proteomic analysis were dipped in liquid nitrogen for quick-freezing immediately with three biological repetitions in each group. Other grouper fillet samples were used for the analysis of quality variation.

### 3.2. Quality Index Analysis

The value of pH was obtained by the pH meter (Sartorius-PB-10, German) according to the method of Shi et al. [11]. An automatic colorimeter (cr-400, Konica Minolta Co., LTD, Shanghai, China) was used to determine the color change of the fish fillets. The value of color difference was calculated as L *(lightness), a * (redness/greenness) and b * (yellowness/blueness) [51]. Dorsal muscles of grouper were used for texture profile analysis including hardness, chewiness, and gumminess. They were cut into 1 cm × 3 cm pieces and tested by the texture analyzer (TA.XT Plus, Stable Micro System, Ltd., English, Guildford). A flat cylindrical probe p/5 (5 mm in diameter) was used to test each sample after two compressions under faceted texture analysis (TPA) mode [52]. Referring to the method of Lu et al. [17] with some modifications, a 2 g sample was put into a tube that contained tissue paper to absorb water and centrifuged (TGL-16A, Changsha, China) at 3000 rpm/min for 5 min at 4 °C. After that, the sample was taken out and weighed. The centrifugal loss was calculated as:

$$\mathrm{Centrifugal}\;\mathrm{loss}\%=\frac{w_{1}-w_{2}}{w_{1}}\times 100$$

where *w*_1_ is the initial weight of the sample and *w*_2_ is the weight after centrifuging the sample.

### 3.3. Protein Extraction and Digestion

To reduce the effect of individual differences, 12 fish were used in this experiment, and the dorsal muscles were mixed evenly and divided into three groups. The samples were taken after 0, 6 and 12 days, respectively, and used to extract the protein. An appropriate amount of sample was suspended on ice in 200 μL lysis buffer (4% sodium dodecyl sulfate (SDS), 150 mM Tris-HCl, and 100 mM dithiothreitol (DTT), pH 7.8). Tissue was disrupted with agitation by a homogenizer and then immediately boiled for 5 min. The samples were further ultrasonicated and boiled again for another 5 min. Undissolved cellular debris was removed by centrifugation at 16,000 rpm for 15 min. The supernatant was collected and quantified with a BCA Protein Assay Kit (Bio-Rad, USA); the protein content of each sample was 10 g. This was followed by SDS-page gel electrophoresis and Coomassie bright blue staining to compare and analyze the protein expression consistency among samples.

As described by Wisniewski et al. [53], digestion of protein (200 μg for each sample) was performed. At first, DTT and other low-molecular-weight components were removed using 200 μL urea (UA) buffer (8 M Urea and 150 mM Tris-HCl, pH 8.0) and repeated ultrafiltration (Microcon units, 30 kD), which was facilitated by centrifugation (12,000 *g*, 10 min). Then, 100 μL 0.05 M iodoacetamide in UA buffer were added to block reduced cysteine residues and the samples were incubated for 20 min in darkness. The filter was washed with 100 μL UA buffer three times and then 100 μL 25 mM NH_4_HCO_3_ twice, and centrifuged at 14,000× *g* for 10 min. Finally, the protein suspension was digested with 4 μg trypsin (Promega) in 40 μL 25 mM NH_4_HCO_3_ overnight at 37 °C, and the resulting peptides were collected as a filtrate. About 150 µg peptides, the concentration of which was determined with OD280 by Nanodrop device, were collected.

### 3.4. TMT Labeling of Peptides and High pH Reverse Phase Fractionation (HPRP)

TMT reagents were used for labeling of peptides according to the manufacturer’s instructions (Thermo Fisher Scientific). Each aliquot (100 μg of peptide equivalent) was reacted with one tube of TMT reagent. Sample labeling was as follows: Group 0-1, 126; Group 0-2, 127 N; Group 0-3, 128 N; Group 6-1, 129 N; Group 6-2, 130 N; Group 6-3, 127 C; Group 12-1, 128 C; Group 12-2, 129 C; and Group 12-3, 130 C. Equal amounts of TMT labeling peptides were mixed in each group, and then, HPRP (Pierce™ High pH Reversed-Phase Peptide Fractionation Kit, Thermo Fisher, Waltham, USA) was used to fractionate peptides after drying. Samples were eventually collected into 15 components. Each component of the peptides was stored at −80 °C for LC-MS analysis.

### 3.5. LC-MS Analysis

The redissolved peptide solution was taken for LC-MS/MS analysis, and each fractional component of the sample was injected once for a total of 15 times for mass spectrometry analysis. The HPLC liquid phase system Easy-nLC was used for separation. Buffer solutions: A, 0.1% formic acid solution; and B, 0.1% formic acid acetonitrile solution (98% acetonitrile). The chromatographic column was balanced with 95% A solution. The sample was loaded onto the chromatographic column, i.e., Trap column (2 cm × 100 μm, 5μm-C18). Then, Thermo Scientific EASY column (75 ms × 120 mm, 3 ms-C18) was separated and the velocity was at 300 nL/min. The relative liquid gradient was as follows: linear gradient of liquid B, 4–7% for 0–2 min; linear gradient of liquid B, 7–20% for 2–67 min; linear gradient of liquid B, 20–35%; linear gradient of liquid B, 30–90% for 79–81 min; liquid B maintained at 90% for 81–90 min. After chromatographic separation, the peptides were analyzed by Q-Exactive Plus mass spectrometer (Thermo Scientific, Waltham, USA) with the following parameters: analysis duration, 90 min; detection method, positive ion; scanning range of parent ion, 300–1800 *m*/*z*. Mass charge ratios of polypeptides and polypeptide fragments were collected as follows: 20 fragment profiles (MS2 scan, HCD) were collected after each full scan. The resolution of the first-level mass spectrometry was 70,000 at *m/z* 200, AGC target was 1e6, and the first-level Maximum IT was 50 ms. The resolution of the second-level mass spectrometry was 35,000 at *m/z* 200, AGC target was 1e5, and the second-level Maximum IT was 50 ms. The MS2 activation type was HCD; the desolation Window was 1.6 Th; and the normalized collision energy was 35.

### 3.6. Protein Database Searching and Analysis

The raw files produced from LC-MS/MS were imported into MaxQuant software (http://www.maxquant.org) (version 1.6.1.0). Then, the transcriptome database was used for data interpretation and protein identification. The title of reference was transcriptome data of *Epinephelus fuscoguttatus* infected by *Vibrio vulnificus*. The protein database was sourced from http://www.ncbi.nlm.nih.gov/sra/SRX3067303. MaxQuant search parameters were set as follows: isobaric labels, TMT 10 plex; reporter mass tolerance, 0.005 Da; max missed cleavages, 2; first search peptide tolerance and MS/MS tolerance, 20 ppm; fixed modifications, carbamidomethyl (C); variable modifications, oxidation (M), acetyl (Protein N-term); and false discovery rate (FDR), <0.01. Razor and unique peptides were used for protein quantification.

### 3.7. Bioinformatics Analysis

Perseus software and R statistical computing software were used to analyze the bioinformatics data [21]. Differentially significant expressed proteins (DSEPs) were screened with the cutoff of a ratio fold-change of >1.20 or <0.83 and *p*-values < 0.05. In our study, DSEPs were compared among Day 0, Day 6 and Day 12 groups according to the filter parameters (PSM, FDR < 0.01, and Protein group FDR < 0.01). Combining the comparative analysis of variance (ANOVA), *t*-test and FDR (Benjamini–Hochberg), all qualitative and quantitative protein analysis results were obtained. Hierarchical clustering was adopted to categorize expression data together according to the protein level.

Huge amounts of data are produced by mass spectrometry technology in proteomics, which represents all the biological processes of the organism. The aim of bioinformatics analysis was to find the source and mechanism for biological changes. UniProt Knowledgebase (UniProtKB)/Swiss-Prot [54], Gene Ontology (GO) enrichment, Kyoto Encyclopedia of Genes and Genomes (KEGG) and protein interaction network analysis were adopted [55].

### 3.8. Statistical Analysis

Data statistical analysis were performed with SPSS software (SPSS, version 19.0). The mean and standard deviations are reported and the *p*-value < 0.05 was considered as statistically significant. Random sampling was used three times in each experiment, and all experiments were replicated three times. Cluster 3.0 and Tree View software packages were used to analyze the hierarchical cluster. Venn diagrams were drawn with R software. Pearson correlations analysis was performed between DSEPs and quality indices (centrifugal loss, pH, L *, a *, b *, hardness, gumminess, and chewiness).

## 4. Conclusions

A TMT-based strategy was applied to elucidate proteins that change in proteomes of grouper fillets during refrigerated storage. A total of 64 differentially significant expressed proteins (DSEPs) were found in the results for the Day 0 vs. Day 6 group comparison and the Day 0 vs. Day 12 group comparison. More proteome changes were found in the Day 0 vs. Day 12 comparison. P06238 (Alpha-2-macroglobulin), Q0IIG5 (ATP-dependent 6-phosphofructokinase), Q3SYR3 (Uncharacterized protein) and Q90YT6 (60S ribosomal protein L32) were potential markers for pH. P51903 (Phosphoglycerate kinase), Q589R5 (Triosephosphate isomerase) and Q90339 (Myosin heavy chain) were closely related with centrifugal loss. The proteins which contributed to the color change were P50143 (T-complex protein 1 subunit gamma), P06238 (Alpha-2-macroglobulin), P20810 (Calpastatin), Q6PHG2 (Hemopexin), P08249 (Malate dehydrogenase) and Q90339 (Myosin heavy chain). Finally, P20810 (Calpastatin), Q90XG0 (Triose phosphate isomerase B), P08249 (Malate dehydrogenase), Q589R5 (Triosephosphate isomerase), Q9I8F9 (Heat shock 70 kDa protein 1), A2ASS6 (Titin), Q90339 (Myosin heavy chain), P23239 (Desmin) and A7E2Y1 (Myosin-7B) were potential markers for texture. This study could help to better understand proteins changes and mechanisms of the quality decline of grouper fillets during refrigerated storage.

## Figures and Tables

**Table 1:** Quality deterioration of grouper fillets during refrigerated storage for 0, 6, and 12 days at 4 °C.

Quality Index	Refrigerated Storage Time (Day)		
	0	6	12
pH	6.79 ± 0.002 ^a^	6.81 ± 0.04 ^a^	6.94 ± 0.04 ^b^
Centrifugal loss%	9.94 ± 1.42 ^a^	17.46 ± 0.86 ^b^	22.76 ± 1.57 ^c^
L *	60.85 ± 0.73 ^a^	55.87 ± 1.49 ^b^	54.43 ± 5.46 ^b^
a *	1.67 ± 0.38 ^a^	2.75 ± 0.70 ^b^	3.77 ± 0.90 ^b^
b *	1.42 ± 0.21 ^a^	3.25 ± 0.67 ^b^	4.05 ± 1.23 ^c^
Hardness	2326.09 ± 152.52 ^a^	1873.28 ± 119.25 ^b^	1245.71 ± 60.28 ^c^
Chewiness	584.74 ± 64.14 ^a^	354.02 ± 33.87 ^b^	163.52 ± 21.33 ^c^
Gumminess	1470.42 ± 78.50 ^a^	738.671 ± 31.97 ^b^	345.27 ± 24.67 ^c^

Data represent the mean ± SD; data on the same row with different letters are significantly different (*p* < 0.05).

**Table 2:** Pearson correlation between differentially significant expressed proteins (DSEPs) and quality indicators of grouper fillets during refrigerated storage (Days 0, 6, and 12) at 4 °C (*p* < 0.05).

Uniprot ID	Description	Classification	pH	Centrifugal Loss	L *	a *	b *	Hardness	Chewiness	Gumminess
P50143	T-complex protein 1 subunit gamma	Binding proteins	−0.769	−0.981	1.000 **	−0.962	−0.997 *	0.926	0.971	0.993
P06238	Alpha-2-macroglobulin	Binding proteins	−0.999 *	−0.985	0.999 *	−0.967	−0.999 *	0.934	0.976	0.820
P20810	Calpastatin	Binding proteins	−0.929	−0.993	0.946	−0.999 *	−0.970	0.997 *	0.997 *	0.981
Q6PHG2	Hemopexin	Binding proteins	−0.827	−0.995	0.994	−0.984	−1.000 *	0.959	0.990	0.985
Q90XG0	Triose phosphate isomerase B	Enzymes	−0.955	−0.980	0.918	−0.993	−0.949	1.000**	0.988	0.963
P08249	Malate dehydrogenase, mitochondrial	Enzymes	−0.862	−0.974	0.984	−0.999 *	−0.999 *	0.975	0.997 *	0.999 *
Q0IIG5	ATP-dependent 6-phosphofructokinase, muscle type	Enzymes	−0.999 *	−0.817	0.679	−0.862	−0.740	0.912	0.842	0.773
P51903	Phosphoglycerate kinase	Enzymes	−0.904	−0.998 *	0.964	−0.801	−0.983	0.991	0.890	0.991
Q3SYR3	Uncharacterized protein	Enzymes	−1.000 *	−0.884	0.768	−0.920	−0.820	0.957	0.904	0.848
Q589R5	Triosephosphate isomerase	Enzymes	−0.868	−1.000 **	0.982	−0.995	−0.935	0.978	0.998 *	0.999 *
Q9I8F9	Heat shock 70 kDa protein 1	Heat shock protein family	0.620	−0.920	0.981	−0.885	−0.961	0.828	0.902	0.996 *
Q08699	40S ribosomal protein S14	Protein turnover	−0.712	−0.962	0.998 *	−0.935	−0.988	0.891	0.948	0.979
Q90YT6	60S ribosomal protein L32	Protein turnover	−0.998 *	−0.903	0.795	−0.936	−0.844	0.969	0.921	0.870
A2ASS6	Titin	Structural proteins	−0.792	−0.988	0.972	−0.972	−0.909	0.999 *	0.999 *	0.996
Q90339	Myosin heavy chain	Structural proteins	−0.892	−1.000 **	0.999	−0.909	−0.999 *	0.998 *	1.000 **	0.995
P23239	Desmin	Structural proteins	−0.933	−0.991	0.942	−0.999 *	−0.968	0.999 *	0.996	0.979
A7E2Y1	Myosin-7B	Structural proteins	−0.946	−0.985	0.929	−0.996	−0.957	1.000 *	0.992	0.971

* Significant correlation (*p* < 0.05). ** Highly significant correlation (*p* < 0.01).

## Supplementary Materials

Figure S1: Interaction network analysis of differentially significant expressed proteins (DSEPs). A (Day 0 vs. Day 6), B (Day 0 vs. Day 12). Table S1: Statistical analysis of protein identification, Table S2: Protein quantification and differential protein analysis, Table S3: Gene Ontology (GO) enrichment analysis for Day 0 vs. Day 6, Table S4: Gene Ontology (GO) enrichment analysis for Day 0 vs. Day 12, Table S5: Kyoto Encyclopedia of Genes and Genomes (KEGG) analysis, Table S6: Pearson correlation between all differentially significant expressed proteins (DSEPs) and quality indicators.
