# Supplementary material for: Analysis of Proteins Associated with Quality Deterioration of Grouper Fillets Based on TMT Quantitative Proteomics during Refrigerated Storage
Source: Molecules. 2019 Jul 20;24(14):2641. doi: 10.3390/molecules24142641 (PMC6680736; doi:10.3390/molecules24142641)
Supplement: Supplementary file 1 [file molecules-24-02641-s001.zip › table S6.docx]

| **Table 2.** Pearson’s correlation between all differentially significant expressed proteins (DSEP) and quality indicators of grouper fillets during refrigerated storage(Day 0, 6, and 12) at 4 °C. | | | | | | | | | | |
| --- | --- | --- | --- | --- | --- | --- | --- | --- | --- | --- |
| **Uniprot ID** | **Description** | **Classification** | **pH** | **Centrifugal loss** | **L*** | **a*** | **b*** | **Hardness** | **Chewiness** | **Gumminess** |
| Q9GLY5 | Inter-alpha-trypsin inhibitor heavy chain H3 | Binding proteins | -0.461 | -0.830 | 0.928 | -0.781 | -0.892 | 0.708 | 0.804 | 0.868 |
| P0DOX8 | Immunoglobulin lambda-1 light chain | Binding proteins | -0.816 | -0.993 | 0.996 | -0.980 | -0.995 | 0.952 | 0.987 | 0.909 |
| P50143 | T-complex protein 1 subunit gamma | Binding proteins | -0.769 | -0.981 | **1.000**** | -0.962 | **-0.997*** | 0.926 | 0.971 | 0.993 |
| Q90WJ9 | Saxitoxin and tetrodotoxin-binding protein 2 | Binding proteins | -0.440 | -0.817 | 0.918 | -0.766 | -0.881 | 0.691 | 0.789 | 0.856 |
| Q9BUR5 | MICOS complex subunit MIC26 | Binding proteins | -0.588 | -0.904 | 0.973 | -0.865 | -0.990 | 0.805 | 0.883 | 0.933 |
| P80191 | Alpha-2-HS-glycoprotein | Binding proteins | -0.774 | -0.983 | 0.985 | -0.964 | -0.995 | 0.929 | 0.973 | 0.994 |
| P06238 | Alpha-2-macroglobulin | Binding proteins | **-0.999*** | -0.985 | **0.999*** | -0.967 | **-0.999*** | 0.934 | 0.976 | 0.820 |
| Q9P4E9 | GTP-binding nuclear protein GSP1/Ran | Binding proteins | -0.962 | -0.975 | 0.908 | -0.990 | -0.941 | 0.909 | 0.984 | 0.957 |
| Q9CY58 | Plasminogen activator inhibitor 1 RNA-binding protein | Binding proteins | -0.816 | -0.993 | 0.996 | -0.980 | -0.896 | 0.953 | 0.987 | 0.991 |
| Q8CI51 | PDZ and LIM domain protein 5 | Binding proteins | -0.963 | -0.587 | 0.407 | -0.652 | -0.484 | 0.731 | 0.624 | 0.527 |
| P20810 | Calpastatin | Binding proteins | -0.929 | -0.993 | 0.946 | -**0.999*** | -0.970 | **0.997*** | **0.997*** | 0.981 |
| P13731 | Serpin H1 | Binding proteins | -0.909 | -0.900 | 0.79 | -0.933 | -0.840 | 0.967 | 0.919 | 0.866 |
| Q6PHG2 | Hemopexin | Binding proteins | -0.827 | -0.995 | 0.994 | -0.984 | **-1.000*** | 0.959 | 0.990 | 0.985 |
| P0DOX6 | Immunoglobulin mu heavy chain | Binding proteins | -0.790 | -0.987 | 0.909 | -0.971 | -0.969 | 0.938 | 0.979 | 0.996 |
| O42249 | Guanine nucleotide-binding protein subunit beta-2-like 1 | Binding proteins | -0.884 | -0.884 | 0.976 | -0.908 | -0.991 | 0.984 | 0.909 | 0.996 |
| Q3MHM5 | Tubulin beta-4B chain | Binding proteins | -0.878 | -0.886 | 0.978 | -0.997 | -0.992 | 0.982 | 0.909 | 0.907 |
| Q8BZ52 | Fibronectin type III | Binding proteins | 0.748 | -0.339 | 0.137 | -0.416 | -0.221 | 0.513 | 0.382 | 0.270 |
| P68246 | Troponin I, fast skeletal muscle | Calcium handling | -0.996 | -0.829 | 0.695 | -0.872 | -0.754 | 0.921 | 0.854 | 0.786 |
| P22316 | Dihydropyridine-sensitive L-type skeletal muscle calcium channel subunit alpha-1 | Calcium handling | 0.689 | 0.952 | -0.995 | 0.924 | 0.983 | -0.876 | -0.937 | -0.972 |
| P13806 | Voltage-dependent calcium channel subunit alpha-2/delta-1 | Calcium handling | -0.226 | -0.666 | -0.806 | -0.601 | -0.752 | 0.510 | -0.630 | 0.718 |
| P54985 | Peptidyl-prolyl cis-trans isomerase | Enzymes | -0.930 | -0.992 | 0.945 | -0.919 | -0.970 | 0.908 | 0.907 | 0.981 |
| Q90XG0 | Triose phosphate isomerase B | Enzymes | -0.955 | -0.980 | 0.918 | -0.993 | -0.949 | **1.000**** | 0.988 | 0.963 |
| P38117 | Electron transfer flavoprotein subunit beta | Enzymes | -0.156 | -0.610 | 0.761 | -0.543 | -0.703 | 0.447 | 0.573 | 0.666 |
| Q2EN81 | ATP synthase subunit O, mitochondrial | Enzymes | -0.910 | -0.907 | 0.961 | -0.887 | -0.981 | 0.993 | 0.990 | 0.989 |
| P80971 | Cytochrome c oxidase subunit 4 isoform 2, mitochondrial | Enzymes | -0.959 | -0.977 | 0.913 | -0.992 | -0.944 | 0.965 | 0.986 | 0.960 |
| Q5R9I5 | Aspartate--tRNA ligase, cytoplasmic | Enzymes | -0.876 | -0.899 | 0.979 | -0.996 | -0.993 | 0.981 | 0.929 | 0.908 |
| Q9DGE0 | Dual specificity mitogen-activated protein kinase kinase 6 | Enzymes | -0.983 | -0.951 | 0.867 | -0.973 | -0.906 | 0.993 | 0.964 | 0.926 |
| P08249 | Malate dehydrogenase, mitochondrial | Enzymes | -0.862 | -0.974 | 0.984 | **-0.999*** | **-0.999*** | 0.975 | **0.997*** | **0.999*** |
| Q6AZA0 | Acetyl-CoA acetyltransferase, mitochondrial | Enzymes | -0.971 | -0.966 | 0.892 | -0.984 | -0.928 | 0.909 | 0.977 | 0.945 |
| Q04467 | Isocitrate dehydrogenase [NADP], mitochondrial | Enzymes | -0.659 | -0.939 | 0.99 | -0.907 | -0.974 | 0.856 | 0.922 | 0.961 |
| Q0IIG5 | ATP-dependent 6-phosphofructokinase, muscle type | Enzymes | **-0.999*** | -0.817 | 0.679 | -0.862 | -0.740 | 0.912 | 0.842 | 0.773 |
| P13619 | ATP synthase F(0) complex subunit B1 | Enzymes | -0.822 | -0.994 | 0.995 | -0.982 | -0.981 | 0.956 | 0.988 | 0.919 |
| P51903 | Phosphoglycerate kinase | Enzymes | -0.904 | **-0.998*** | 0.964 | -0.801 | -0.983 | 0.991 | 0.890 | 0.991 |
| Q32KV0 | Phosphoglycerate mutase | Enzymes | -0.714 | -0.880 | 0.763 | -0.917 | -0.816 | 0.955 | 0.901 | 0.844 |
| O57656 | Glycerol-3-phosphate dehydrogenase [NAD(+)] | Enzymes | -0.963 | -0.974 | 0.907 | -0.990 | -0.939 | 0.929 | 0.984 | 0.955 |
| Q3SYR3 | Uncharacterized protein | Enzymes | **-1.000*** | -0.884 | 0.768 | -0.920 | -0.820 | 0.957 | 0.904 | 0.848 |
| Q4R5B0 | ATP synthase subunit gamma, mitochondrial | Enzymes | -0.763 | -0.980 | 0.866 | -0.959 | -0.997 | 0.923 | 0.970 | 0.992 |
| O13276 | L-lactate dehydrogenase A chain | Enzymes | -0.883 | -0.900 | 0.976 | -0.907 | -0.991 | 0.984 | 0.909 | 0.996 |
| P00348 | Hydroxyacyl-coenzyme A dehydrogenase, mitochondrial | Enzymes | -0.542 | -0.879 | 0.959 | -0.836 | -0.931 | 0.771 | 0.857 | 0.911 |
| Q589R5 | Triosephosphate isomerase | Enzymes | -0.868 | **-1.000**** | 0.982 | -0.995 | -0.935 | 0.978 | **0.998*** | **0.999*** |
| Q5MJ86 | Glyceraldehyde-3-phosphate dehydrogenase 2 | Enzymes | -0.949 | -0.984 | 0.926 | -0.995 | -0.955 | 0.855 | 0.991 | 0.969 |
| B5DGM7 | Fructose-bisphosphate aldolase A | Enzymes | -0.962 | -0.975 | 0.909 | -0.990 | -0.941 | 0.994 | 0.984 | 0.957 |
| P11607 | Sarcoplasmic/endoplasmic reticulum calcium ATPase 2 | Enzymes | 0.867 | 0.522 | -0.334 | 0.591 | 0.413 | -0.675 | -0.559 | -0.459 |
| P10895 | 1-phosphatidylinositol 4,5-bisphosphate phosphodiesterase delta-1 | Enzymes | 0.994 | 0.924 | -0.825 | 0.953 | 0.870 | -0.980 | -0.940 | -0.894 |
| Q38HM4 | E3 ubiquitin-protein ligase TRIM63 | Enzymes | 0.856 | 0.909 | -0.986 | 0.992 | 0.997 | -0.972 | -0.996 | -0.991 |
| P15122 | Aldo-keto reductase family 1 member B1 | Enzymes | 0.607 | 0.914 | -0.978 | 0.877 | 0.957 | -0.819 | -0.985 | -0.941 |
| Q90474 | Heat shock protein HSP 90-alpha 1 | Heat shock protein family | 0.566 | -0.892 | 0.966 | -0.851 | -0.941 | 0.789 | -0.871 | 0.922 |
| Q9I8F9 | Heat shock 70 kDa protein 1 | Heat shock protein family | 0.620 | -0.920 | 0.981 | -0.885 | -0.961 | 0.828 | 0.902 | **0.996*** |
| Q08699 | 40S ribosomal protein S14 | Protein turnover | -0.712 | -0.962 | **0.998*** | -0.935 | -0.988 | 0.891 | 0.948 | 0.979 |
| Q90YS3 | 40S ribosomal protein S2 | Protein turnover | -0.628 | -0.924 | 0.983 | -0.889 | -0.964 | 0.834 | 0.906 | 0.949 |
| Q5E958 | 40S ribosomal protein S8 | Protein turnover | -0.464 | -0.832 | 0.929 | -0.783 | -0.894 | 0.710 | 0.806 | 0.870 |
| Q90YT6 | 60S ribosomal protein L32 | Protein turnover | **-0.998*** | -0.903 | 0.795 | -0.936 | -0.844 | 0.969 | 0.921 | 0.870 |
| Q9W6X9 | 40S ribosomal protein S24 | Protein turnover | -0.670 | -0.944 | 0.992 | -0.913 | -0.977 | 0.863 | 0.928 | 0.965 |
| A2ASS6 | Titin | Structural proteins | -0.792 | -0.988 | 0.972 | -0.972 | -0.909 | **0.999*** | **0.999*** | 0.996 |
| Q90339 | Myosin heavy chain | Structural proteins | -0.892 | **-1.000**** | 0.999 | -0.909 | **-0.999*** | **0.998*** | **1.000**** | 0.995 |
| P23239 | Desmin | Structural proteins | -0.933 | -0.991 | 0.942 | **-0.999*** | -0.968 | **0.999*** | 0.996 | 0.979 |
| A7E2Y1 | Myosin-7B | Structural proteins | -0.946 | -0.985 | 0.929 | -0.996 | -0.957 | **1.000*** | 0.992 | 0.971 |
| A2AAJ9 | Obscurin | Structural proteins | -0.919 | -0.995 | 0.954 | -0.942 | -0.976 | 0.995 | 0.909 | 0.986 |
| Q4V8J7 | Spindlin-1 | Miscellaneous | -0.747 | -0.974 | 0.985 | -0.952 | -0.995 | 0.913 | 0.964 | 0.988 |
| P79819 | Serotransferrin | Miscellaneous | -0.810 | -0.992 | 0.997 | -0.978 | 0.855 | 0.949 | 0.985 | 0.909 |
| N310_c0_g1\|m.6737 | Uncharacterized protein | Miscellaneous | -0.955 | -0.980 | 0.918 | -0.993 | -0.948 | 0.905 | 0.988 | 0.963 |
| P68103 | Elongation factor 1-alpha 1 | Miscellaneous | -0.784 | -0.986 | 0.909 | -0.968 | -0.919 | 0.935 | 0.977 | 0.995 |
| Q3SZ54 | Eukaryotic initiation factor 4A-I | Miscellaneous | -0.600 | -0.910 | 0.976 | -0.873 | -0.954 | 0.814 | 0.891 | 0.938 |
| P50580 | Proliferation-associated protein 2G4 | Miscellaneous | -0.842 | 0.480 | -0.288 | 0.551 | 0.369 | -0.639 | -0.518 | -0.415 |
| C0HL13 | Low-density lipoprotein receptor-related protein 2 | Miscellaneous | -0.988 | 0.793 | -0.650 | 0.841 | 0.712 | -0.895 | -0.819 | -0.747 |
