# Supplementary figures and images for: Analysis of Proteins Associated with Quality Deterioration of Grouper Fillets Based on TMT Quantitative Proteomics during Refrigerated Storage
Source: Molecules. 2019 Jul 20;24(14):2641. doi: 10.3390/molecules24142641 (PMC6680736; doi:10.3390/molecules24142641)

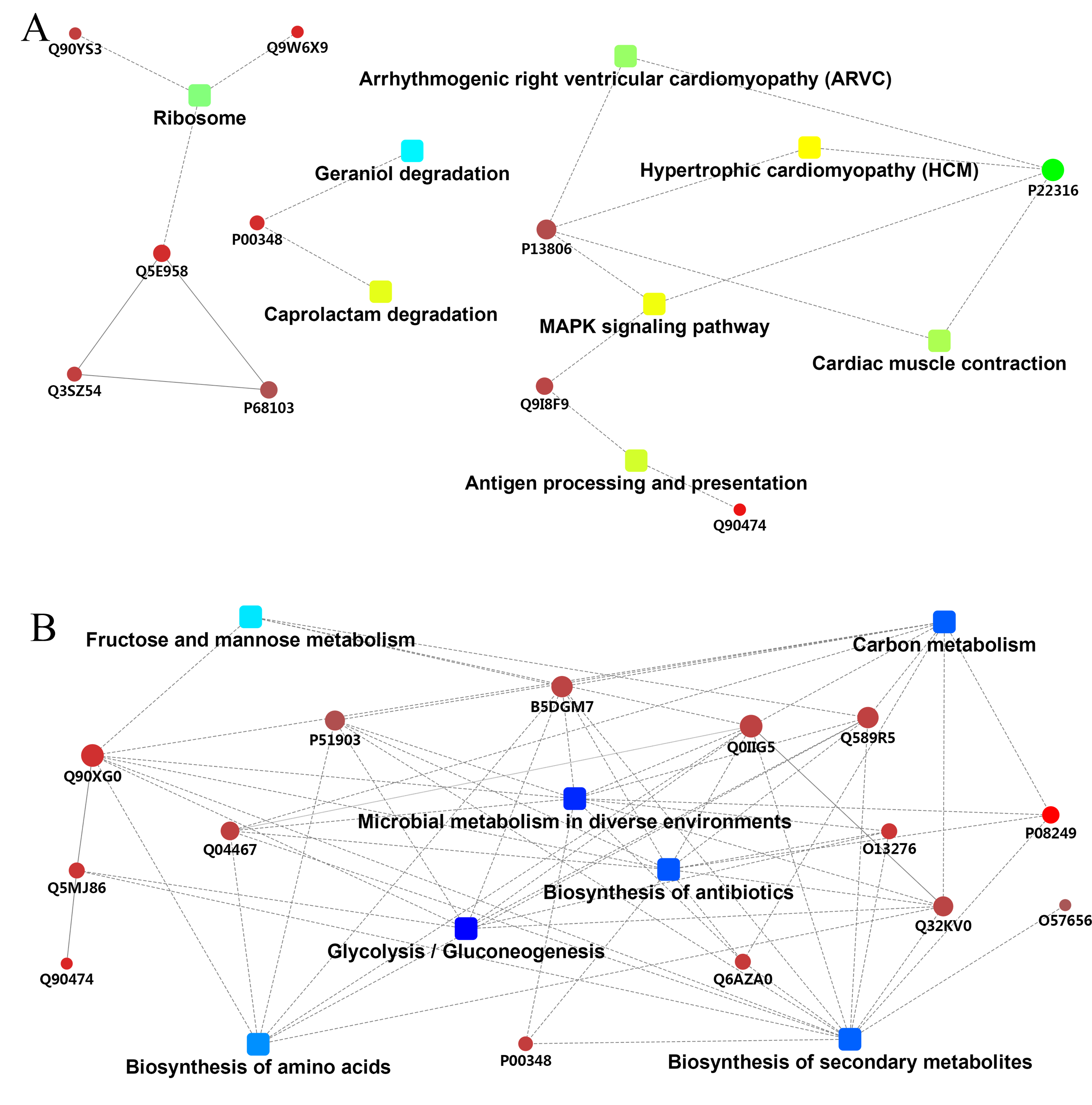

Supplement: Supplementary file 1 [file molecules-24-02641-s001.zip › Figure S1..tif]
